# The pharmacologist’s fertility

**DOI:** 10.3325/cmj.2013.54.308

**Published:** 2013-06

**Authors:** Thomas J. Papadimos

**Affiliations:** Department of Anesthesiology, The Ohio State University Wexner Medical Center, Columbus, OH, USA

All that was left were ashes, which were placed about the base of a family tree in the backyard. I often think about those ashes. Those bits of carbon carried such a biological bang, a compaction of scholarly excellence and a cosmic distillation of intellect.

**Figure 1:**
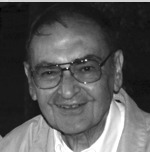
Professor Vladimir Nigrovic

From time to time it is worth pausing and remembering those who have been our teachers and mentors. In this instance I honor and remember Vladimir Nigrovic – a Croatian physician who was a gentleman, a scholar, and a widely recognized authority on diuretics and neuromuscular blockade (Figure 1). He was a teacher and mentor who made a difference in my life and to those in the Anesthesiology and Pharmacology Departments at the University of Toledo College of Medicine, USA. His passion and enthusiasm were infective, and the scope of his intellect was inspiring. He is missed and fondly remembered.

Dr Vladimir Nigrovic was born on March 3, 1934 in the city of Sarajevo, but grew up in Zagreb, which at that time was in the kingdom of Yugoslavia. He always liked the stars. He wanted to be an astronomer, but World War II broke out and the Balkans became a bit less friendly. Many were victimized, and maybe that is why the stars held less interest for him, maybe they dimmed a bit during those difficult days.

He grew into a lanky fellow, good humored and quick-witted. He turned his interests to the curative sciences. Now in a post world war nuclear society, his thoughts drifted toward the unthinkable, causing him to redouble his efforts regarding the art of critical thinking in order to contemplate, through thoughtful reflection, solutions to all things catastrophic and calamitous. This inquisitive youth, who had a penchant for fine tobacco and asking the difficult, pithy interrogatives that confront society, went on to study medicine in Zagreb and then Heidelberg, Germany where he received his medical degree in 1962 (and met his bride to be, Elizabeth, they eventually had three children). He developed a keen interest in pharmacology. Soon thereafter his scientific deliberations resulted in his discovery of how to remove radioactive cesium, a component of nuclear fallout, from the human body (1,2). What an incredible and insufficiently recognized accomplishment! Thereafter, he made his way to the United States where he focused his intellectual skills on the mechanisms of action of diuretics and muscle relaxants. He became a founding member of the Department of Pharmacology at the Medical College of Ohio (now the University of Toledo College of Medicine) in 1968. His interest in drug interactions and mechanisms of action sent him back to clinical medicine as a resident in Anesthesiology in 1977, and he stayed on as a faculty member. He went on to become a Professor of Pharmacology and Anesthesiology. His scholarly work in diuretics and neuromuscular blockers was notable; he had over 100 publications. He went on to receive his university’s highest honor for teaching, and was twice awarded the B.B. Sankey Award for progress in Anesthesiology.

After his retirement in 1997, until his death, he came to his former place of work every day. He donned his scrubs and cap and went to work on his computer, hashing out regressions, decay constants, volumes of distribution, mechanisms of action, writing letters to editors, pursuing lines of inquiry that only those whose brains were on rocket fuel twenty four hours a day could understand and keep up with…and he mentored. Ah yes, his mentoring. He always had time to mentor and teach. He had a patient gaze that pierced your eyes as you posed the interrogative, and a Socratic response that left you with more questions than answers. Of course, all he was doing was trying to get you to find the light. He did not mind lighting the torch to help you get out of the cave, but he preferred that you lit the torch yourself, or at least identified an “enlightened beginning” on your own, thereby allowing you to navigate the experience. You see, he was not training residents to be anesthesiologists; he was training students, residents, and junior faculty members to be future mentors and teachers. He encouraged us to question everything that was dogma, everything we thought to be sacred, and to have no fear in seeking or telling the truth. He made us grow by teaching us *parrhesia…* speaking truth to power (3). In fact, he insisted that *parrhesia* was an obligation, that is, to speak freely at your own personal risk for the common good (as did the ancient Greeks and Michel Foucault). We were all his seedlings and he tilled the fields of our academic growth to the best of his ability in order to ensure that we were well rooted in our field of study, not only scientifically, but also politically and socially as well.

Alas, he fell ill. One day he walked into my office and sat down in the chair opposite me. A place he frequented on many occasions where we solved, or at least complained about, the problems of the world. “Tom,” he said in his heavy accent, “I can no longer think. I cannot believe I cannot think.” His now ashen appearance was haunting. His eyes, those windows to the soul, were hollow, and his attempts to catch his breath made me flinch. The small beads of perspiration on his forehead, soaking his surgical cap, like so many tears foretelling an impending tragic ending, rolled down endlessly. I felt as if my head would burst. My friend, mentor, and teacher was dying of cancer and there was nothing I, he, or anyone else could do about it. He frightened me because he mirrored my own inevitable mortality, and he made me despair because it was obvious that I was powerless to cure him, to comfort him, or to temper his anguish. His had been a sharp, meticulous, stellar mind; sometimes little appreciated by his critics, but dearly loved by all those near to him.

He got up from the chair and said, “I think I must go.” And he did. “Vlado,” as we fondly referred to him, passed away on September 4, 2008. As we approach the fifth anniversary of his death, many of us will pause and take a moment to reflect on his accomplishments, fellowship, mentorship, and friendship. He came and went; a giant wind-like gust of intellect that brushed us all with grace and goodness. He was not just a scientist and physician, he was a great storyteller, a lover of the arts and of letters; he was a well-read “big idea” type of gentleman and scholar. His world was large. It had room for lots of people and differing points of view. His acquaintance left our academic and personal landscapes a little more fertile, and the world a little less arid.
